# Melamine Recognition: Molecularly Imprinted Polymer for Selective and Sensitive Determination of Melamine in Food Samples

**DOI:** 10.1155/2020/8864144

**Published:** 2020-11-01

**Authors:** Mohadese Biabani, Azizollah Nezhadali, Ahmad Nakhaei, Hossein Nakhaei

**Affiliations:** ^1^Department of Chemistry, Payame Noor University, P.O. Box 19395–4697, Tehran, Iran; ^2^Young Researchers and Elite Club, Mashhad Branch, Islamic Azad University, Mashhad, Iran; ^3^Health Promotion Research Center, Zahedan University of Medical Sciences, Zahedan, Iran

## Abstract

In this study, a sensitive and selective sensor is constructed to measure the melamine (MEL) using molecular imprinting polymer (MIP) technique. Chemical and electrochemical techniques are used to construct the MIP and quantitative measurements. The constructed sensor was modified with GO-Fe_3_O_4_@SiO_2_ nanocomposite. Screening and optimization of factors are done using statistical methods, including Plackett–Burman design (PBD) and central composite design (CCD). Under the optimized conditions, an MIP sensor showed a linear range from 5.0 × 10^−7^ to 1.0 × 10^−5^ M MEL concentration with a correlation coefficient (*R*^2^) of 0.9997. The limit of detection was obtained (0.028 *µ*M) with a highly reproducible response (RSD 2.15%, *n* = 4). The electrochemical sensor showed good results for the determination of MEL in food samples.

## 1. Introduction

**Scheme 1:**
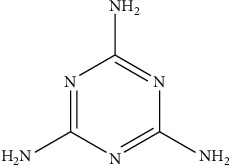
Structure of melamine (MEL).

Melamine (MEL) (Scheme 1), triamino triazine, is an organic compound with the formula C_3_H_6_N_6_, which contains 67% nitrogen by mass. MEL can be combined with formaldehyde and other agents to produce melamine resins. Such resins are characteristically durable thermosetting plastic used in high-pressure decorative laminates and dry erase boards. MEL foam is used as insulation, soundproofing material, and polymeric cleaning products. Besides, it is used in adhesives, paints, permanent-press fabrics, textile finishes, tarnish inhibitors, paper coatings, and fertilizer mixtures [1]. Because it has protein-like properties, it is sometimes illegally added to food products like pet foods, liquid milk, yogurts, egg, frozen desserts, powdered milk, cereal products, confectionaries, cakes and biscuits, protein powders, and some processed foodstuffs to increase the apparent protein content. Ingestion of MEL may lead to reproductive damage, or bladder or kidney stones, and bladder cancer. It is also an irritant when inhaled or in contact with the skin or eyes. The United Nations' food standards body has set the maximum amount of MEL allowed in powdered infant formula to 1 mg/kg and the amount of the chemical allowed in other foods and animal feed to 2.5 mg/kg. While not legally binding, the levels allow countries to ban the importation of products with excessive levels of MEL [2–4].

Various analytical methods are described for the determination of very low amounts of MEL in different samples, including gas chromatography [5], capillary zone electrophoresis [6], high-performance liquid chromatography [7], and reversed-phase high-performance liquid chromatography with solid-phase extraction [8]. Although these methods have been successfully employed, they are expensive and time-consuming and require high skill to use. On the contrary, electrochemical methods are simple, fast, inexpensive, and useful research tool for investigating a large range of molecules, and they can be an interesting alternative to other instrumental techniques. MEL is a nonelectroactive molecule, but using some electroactive materials, called probes, electrochemical methods can be used to measure MEL [9]. [Fe (CN)_6_]^3−^/[Fe (CN)_6_]^4−^ solution is a common electrochemical indicator for nonelectroactive molecules [10].

One of the effective separation methods that have appeared in recent years is molecularly imprinting polymers (MIPs). MIP is synthesized by simultaneous polymerization of functional and cross-linking monomers in the presence of the template molecule and used as a powerful, sensitive, and selective absorber for the identification and measurement of the template molecule. MIPs have several advantages, including low cost, good physical and chemical stability, high selectivity, and simplicity [11–15]. MIPs have been widely used in solid-phase extraction [16], chromatographic separation [17], drug release [18], reaction catalysts [19], enzyme mimics [20], cancer biomarkers and viruses [21], and sensors [22–24]. Precipitation polymerization for producing MIPs is more popular because, in this method, regular shape MIP beads are obtained, and the polymeric chains are grown individually to microspheres. In addition, this method does not need the porogen agents and has easy and fast procedure. Because of fantastic advantages of chemical methods for constructing MIP and electrochemical methods for determination, their combination can be a smart tool to achieve a new selective and sensitive technique [25–27]. Pyrrole is an interesting functional monomer because it is suitable in a natural pH range, and its polymer is made easily with a high chemical and electrochemical stability [28–30]. Low conductivity is the most common problem of MIP sensors that lead to decrease in electron transfer and low sensitivity. It is because of forming a thick polymeric film on the surface of electrode. The sensitivity and performance of the sensor are enhanced by emerging MIP with nanoparticles [31] that this construction leads to the high surface-to-volume ratio, which results in an increase in surface area per weight unit of polymer. In addition, due to the geometric characteristics of MIP/nanoparticles, the penetration of the target molecule into the polymer cavities is increased, resulting in a faster mass transfer rate [32–36]. Nanomaterials such as carbon nanotubes (CNTs), graphene oxide (GO), and metal oxide nanoparticles such as ZnO, Fe_3_O_4_, CuS, SiO_2_, and TiO_2_ are suitable to improve the selectivity and sensitivity of MIPs. In addition, core-shell nanostructures are a kind of new nanomaterials. In these nanostructures, one nanoparticle is coated by another nanoparticle. Many properties of core-shell nanoparticles are more efficient and improved than single nanoparticles. Due to the unique properties of these nanostructures including the unique mechanical, optical, and thermal properties, use of core-shell nanoparticles has been increasing in recent years [37, 38]. In these structures, the shell is used to protect the core particles from physical and chemical changes. Another potential purpose of the shell is to improve the activity of the core particle surface as well as stability and scattering. Through surface coating, core particles can have magnetic, optical, and catalytic properties that are unique to shell particles [39–41]. In the present work, to take advantage of the core-shell nanoparticles, using the sol-gel method, the surface of the Fe_3_O_4_ nanoparticles deposited on GO nanoparticles was coated with a layer of SiO_2_ nanoparticles, and finally, the GO-Fe_3_O_4_@SiO_2_ nanocomposite was made.

In this study, an electrochemical sensor was developed for the determination of MEL. First, chemical polymerization of pyrrole was carried out in the presence of MEL, and then, by removing the MEL from the MIP holes, it deposited on the bare of Pt electrodes to construct the electrochemical sensor as a selective and sensitive microsolid-phase preconcentration sensor to the determination of MEL. Multivariate techniques including Plackett–Burman design (PBD) and central composition design (CCD) were used for screening and optimization of the factors affecting the performance of extraction and determination of MEL, respectively.

## 2. Results and Discussion

Cyclic voltammetry (CV) is a reversible electrochemical technique used to study the electrochemical behavior of electroactive species. However, for nonelectroactive species, some electroactive materials are used as probes. The solution of Fe (CN)_6_^4−^ and Fe (CN)_6_^3−^ is one of the most popular probes used in the analytical process [42]. In the present work, the oxidation current of MIP/GO-Fe_3_O_4_@SiO_2_/Pt electrode as a working electrode in 0.3 M solution of [Fe (CN)_6_]^4−^ and [Fe (CN)_6_]^3−^ was measured before loading the electrode in MEL standard solution (*I*_*p*_). After loading the MIP/GO-Fe_3_O_4_@SiO_2_/Pt electrode with the standard solution of MEL, the oxidation current (I_MIP_) of 0.3 M solution of [Fe (CN)_6_]^4−^ and [Fe (CN)_6_]^3−^was measured and the oxidation current decrease (∆*I* = *I*_*p*_–*I*_MIP_) was calculated as the amount of the MEL molecules trapped in the imprinted polymer holes.

### 2.1. Experimental Design

#### 2.1.1. Screening of Significant Factors

To maximize the amount and accuracy of information that was received from a given set of experimental runs, a planned sequence of experiments linking changes in input variables with changes in ∆*I* was designed. This experimental design facilitated the study of how responses change and interact at different variable settings. The PBD design, as a great value in screening experiments, identifies the effective factors and reduces the number of runs [43]. In the present work, the nine factors were chosen for the investigation, which are the amount of GO-Fe_3_O_4_@SiO_2_ (g) (A), [PY]/[MEL] (B), extraction solvent (C), the amount of FeCl_3_ (g) (D), stirring rate of polymerization solution (r.p.m.) (E), the amount of MIP/GO-Fe_3_O_4_@SiO_2_ (g) (F), the polymerization time (h) (G), stirring rate of loading solution (r.p.m) (H), and loading time (min) (J). A low and high level was considered for each of the variables. A PBD design was carried out for nine factors, consisting of 12 randomized runs.  Table 1 shows the experimental results for the 12-run PBD design.

**Figure 1:**
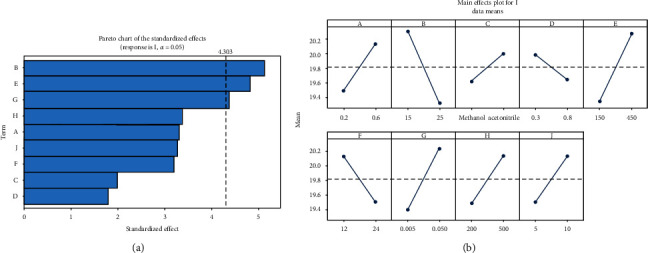
(a) The Pareto plot; (b) the main effect plot.

Figures 1(a) and 1(b) illustrate the standardized Pareto plot of the main effects for PBD design and main effect plot for voltammetric response at 95% confidence level (*p* ≤ 0.05), respectively. The Pareto plot shows that effects of B, E, and G factors are most important to the analytical process. The main effects plot for ∆*I* shows the effective level of each factor. Therefore, B factor in the low level and E and G factors in the high level have more impact on the experiment and need to be optimized more accurately.

#### 2.1.2. Optimization

CCD is a simple and useful design used to optimize a wide range of empirical effective factors [44]. A three-level CCD with 20 runs was carried out for optimization of the process after screening by PBD design. The results are shown in Table 2 for each experiment.

The aim of this analysis is to increase the ∆*I*, which is a measure of the amount of the MEL molecules trapped in imprinted polymer holes. The following equation was obtained based on the regression analysis:

(1) $$\begin{array}{c} \Delta I=-13.96+4.138\text{B}-0.00837\text{E}+370.2\text{G}-0.11227{\text{B}}^{2}+0.000007{\text{E}}^{2}+1933{\text{G}}^{2}+0.001040\text{BE}-25.525\text{BG}-0.2758\text{EG}. \end{array}$$

Using analysis of variance (ANOVA), presented in Table 3, the validation of the statistical result was analyzed. *R*^2^ and *R*^2^_adj_ for models were obtained as 99.29% and 98.65% (*p* ≤ 0.05), respectively. The lack-of-fit *P* value was obtained as 0.171. According to the response surface optimization, the optimal conditions were obtained as 11.8 and 300 r.p.m. and 0.07 g for B, E, and G factors, respectively.

#### 2.1.3. Surface Characterization

**Figure 2:**
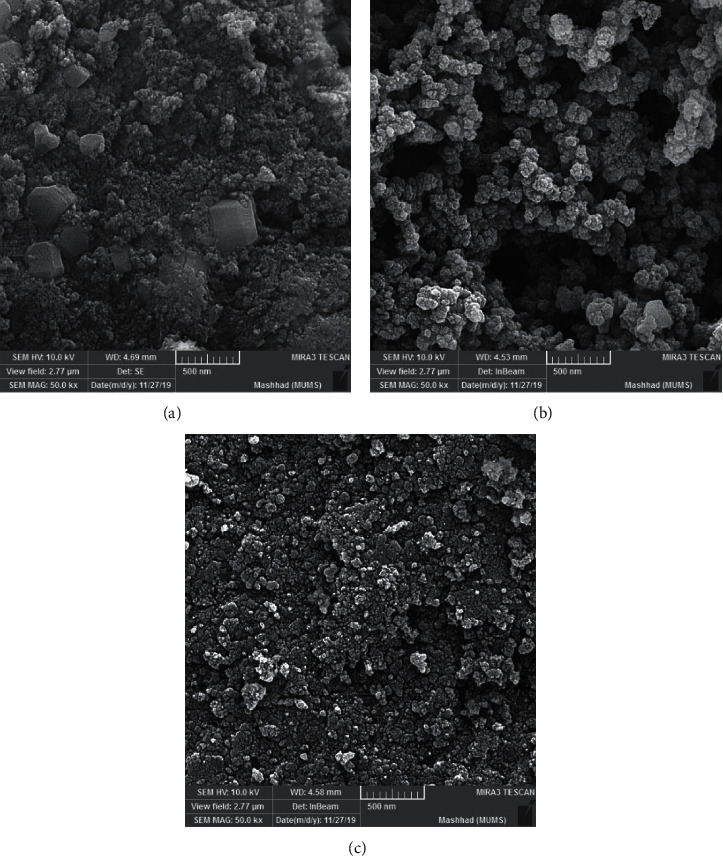
The SEM image of (a) GO-Fe_3_O_4_@SiO2, (b) MIP/GO-Fe_3_O_4_@SiO_2_, (c) and NIP/GO-Fe_3_O_4_@SiO_2_.

**Figure 3:**
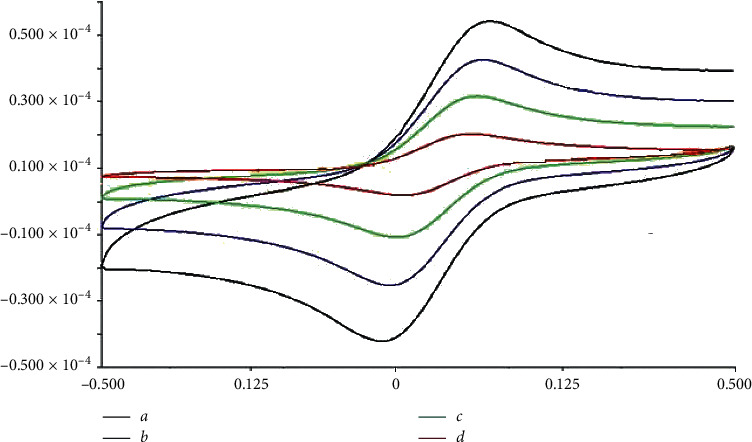
The CV voltammograms of the 0.3 M probe solution at the surface (a) unloaded MIP/GO-Fe_3_O_4_@SiO_2_/Pt, (b) loaded NIP/GO-Fe_3_O_4_@SiO_2_/Pt, (c) loaded MIP/Pt, and (d) loaded MIP/GO-Fe_3_O_4_@SiO_2_/Pt.

The morphological structures of GO-Fe_3_O_4_@SiO_2_ nanocomposite (Figure 2(a)), MIP/GO-Fe_3_O_4_@SiO_2_ (Figure 2(b)), and NIP/GO-Fe_3_O_4_@SiO_2_ (Figure 2(c)) were investigated by scanning electron microscopy (SEM).  Figure 3 shows image of the amorphous GO-Fe_3_O_4_@SiO_2_ nanocomposite at a magnification of 50000. Figure 2(b) shows a uniform imprinted polymeric film of a network of holes that has spread to the surface of the GO-Fe_3_O_4_@SiO_2_ nanocomposite. The SEM images (Figures 2(b) and 2(c)) demonstrate the significant morphological difference between MIP/GO-Fe_3_O_4_@SiO_2_ and NIP/GO-Fe_3_O_4_@SiO_2_, respectively. The polymerization conditions, such as the type of [PY]/[MEL], polymerization solvent, polymerization time, are effective on imprinted polymer [45]. The SEM imaging of MIP clearly shows an irregular morphology that facilitates the fast binding of template molecules to the polymer [46]. The surface of MIP/GO-Fe_3_O_4_@SiO_2_ exhibits more porosity than that of NIP/GO-Fe_3_O_4_@SiO_2_ (Figures 2(b) and 2(c)). The presence of the MEL in the polymerization solution causes the formation of holes in the MIP film and changes the morphology of the polymer [47].

#### 2.1.4. The Molding Effect

To investigate of the presence of holes on the surface of MIP, the CV voltammograms of the 0.3 M probe solution at the surface unloaded MIP/GO-Fe_3_O_4_@SiO_2_/Pt (a), loaded NIP/GO-Fe_3_O_4_@SiO_2_/Pt (b), loaded MIP/Pt (c), and loaded MIP/GO-Fe_3_O_4_@SiO_2_/Pt (d) in the potential range of −0/500 to 0/500 V were investigated. The results are shown in Figure 3. For this purpose, the MIP/GO-Fe_3_O_4_@SiO_2_/Pt sensor was manufactured according to the optimum conditions. Before loading MEL, it was immersed in the 0.3 M probe solution, and its cyclic voltammogram was recorded (a). Then, the sensor was loaded under optimum conditions, and the cyclic voltammogram of 0.3 M probe solution was again recorded (d). By comparing (a) and (d), it can find that when the MIP cavities are empty, Fe^2+^ and Fe^3+^ ions can easily penetrate to the electrode surface and are oxidized and resuscitated, but the sharp decrease in the current in (d) shows the blocked cavities after loading the sensor well. The cavities created during the polymerization process are completely consistent with the MEL molecules in terms of shape, size, and functional groups. By comparing (d) and (b), the molding effect is well known. In other words, during the polymerization process, the complex of PY-MEL is formed because of hydrogen bonds between the N-H group of PY monomers and NH_2_ group of MEL molecules. As a result, the MEL molecules are trapped due to the formation of hydrogen bonds with the PY monomers in the polymer tissue, which increases the porosity of the MIP. Therefore, after loading, the penetration of Fe^2+^ and Fe^3+^ ions into the electrode surface will be less than NIP. Comparison of (d) and (c) shows the modification of MIP with GO-Fe_3_O_4_@SiO_2_ nanocomposite and demonstrates the role of GO-Fe_3_O_4_@SiO_2_ nanocomposite in increasing the polymer surface area. In other words, GO-Fe_3_O_4_@SiO_2_ forms a mediating layer between the MIP and the surface of the Pt electrode and increases the surface area of the electrode for electrochemical processes. In addition, it increases the conductivity of the electrode and facilitates the electron transfer process at the surface of the modified electrode [48].

#### 2.1.5. Figures of Merit

**Figure 4:**
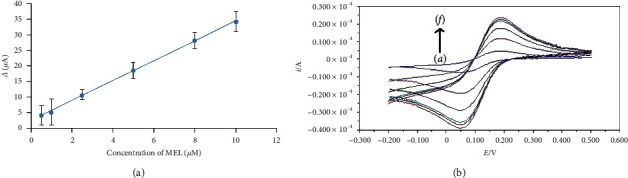
(a) The calibration curve of MEL in different concentrations and (b) the cyclic voltammograms of the MIP/GO-Fe_3_O_4_@SiO_2_/Pt electrode after loading in different solutions (*a*: 8 *μ*M, *b*: 5 *μ*M, *c*: 2.5 *μ*M, *d*: 1 *μ*M, *e*: 0.5 *μ*M, and *f*: 0.1 *μ*M) of MEL in 0.3 M probe solution.

In order to investigate the dependence of the analytical response of the proposed sensor on the concentration of MEL, different concentrations of MEL in optimal conditions were measured by the proposed MIP/GO-Fe_3_O_4_@SiO_2_/Pt electrode. The calibration curve showed a dynamic linear range from 5.0 × 10^−7^ to 1.0 × 10^−5^ M MEL (Figure 4), with a linear regression equation:

(2) $$\begin{array}{c} \Delta I=2.5638+3.2143C_{\text{MEL}},\\ R^{2}=0.9997, \end{array}$$

where *C*_MEL_ is the MEL concentration and ∆*I* (*µ*A) is the difference of *I*_*p*_ and *I*_MIP_ voltammetric anodic peak current. The correlation coefficient is 0.9997. The detection limit of MEL was obtained as 0.028 *µ*M. The repeatability of the MIP/GO-Fe3O4@SiO2/Pt electrode was investigated, and the ∆*I* was determined using the same electrode. The repeatability and reproducibility of the MIP/GO-Fe3O4@SiO2/Pt electrode were performed with repeated measurements of the same sensor in one day and repeated measurements with different sensors, respectively. Relative standard deviations (RSD%) of 2.15% (*n* = 4) and 6.43% (*n* = 4) were obtained for repeatability and reproducibility, respectively. Interday stability of the sensor was investigated, and the current response was measured; the current was unaltered, and a decrease of 9.27% in the current response occurred after the 4th day. These results indicate that the electrode has an acceptable reproducibility and long-term stability, which make it attractive for fabrication of electrochemical sensors.

#### 2.1.6. Selectivity of MIP/GO-Fe3O4@SiO2/Pt Electrode

In order to evaluate the proposed sensor selectivity, the ∆*I* was investigated for the solution containing 2.0 × 10^−6^M MEL and different concentrations of each interfering molecules such as arginine, galactose, glucose, maltose, rabeprazole, and fluvoxamine.  Table 4 shows the results of measurement of MEL in the presence of interfering molecules. The results confirm the selectivity of the MIP/GO-Fe_3_O_4_@SiO_2_/Pt electrode for the MEL relative to interfering substances.

#### 2.1.7. Analysis of Food Samples

**Figure 5:**
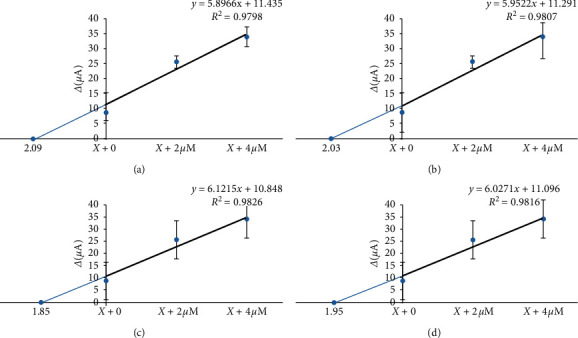
The calibration curve for (a) milk, (b) yoghurt, (c) cheese, and (d) dough.

The method of standard addition is a type of quantitative analysis approach often used in analytical chemistry whereby the standard is added directly to the aliquots of analyzing samples. This method is used in situations, where the sample matrix also contributes to the analytical signal, a situation known as the matrix effect, thus making it impossible to compare the analytical signal between sample and standard using the traditional calibration curve approach. In the present procedure, three solutions containing 2 *µ*M of MEL and different amounts of standard solutions of MEL (0, 2, and 4) were prepared and diluted to 10 mL with deionized water. Then, the proposed sensor was used for preconcentration and determination of MEL.  Figure 5 shows the calibration curve for any food sample.  Table 5 shows the results of measurement of MEL in milk, yoghurt, cheese, and dough samples. Each analysis was repeated three times under the optimized conditions. The recovery (%) for the analyzed food samples showed good results (92.5–104.5%).

## 3. Experimental

### 3.1. Chemicals and Reagents

Iron(III) chloride (99–102%), iron(II) chloride (99.9999 Suprapur), hydrochloride acid (37%), ammonia (99.5%), pyrrole (≥97%), methanol (99.9%), acetic acid (99.5%), and ethanol (85%) were purchased from Merck (Darmstadt, Germany). Potassium thiocyanate (99%), potassium hexacyanoferrate (III) (99%), potassium hexacyanoferrate (II) trihydrate (99.95%), melamine (99%), and sodium hydroxide (98%) were purchased from Sigma-Aldrich. GO nanoparticles (>95%) and SiO_2_ (˃99%) nanoparticles were purchased from Iranian Nanomaterials Pioneers Co. (Mashhad, Iran).

### 3.2. Apparatus

The electrochemical studies were done with a three-electrode system: a MIP/GO-Fe_3_O_4_@SiO_2_/Pt, a platinum wire, and an Ag/AgCl (saturated KCl) as the working electrode, the counterelectrode, and the reference electrode, respectively. The voltammetric measurements were carried out by Autolab PGSTAT 12 potentiostat-galvanostat (Ecochemie, The Netherlands). The surface evaluations of sensors were performed by scanning electron microscopy (SEM) in an Oxford S360 SEM (Britain) microscope. The sonication of GO-Fe_3_O_4_@SiO_2_ was performed using a Hielscher ultrasonic bath processor (UTR200, Germany). Duo to shake of the extraction columns containing polymer and loading solution during the extraction process, Shaker KS130 EKE (Germany) was used.

#### 3.2.1. Synthesis of GO-Fe_3_O_4_@SiO_2_ Nanocomposite

GO-Fe_3_O_4_@SiO_2_ nanocomposite was prepared in two steps. In the first step, GO-Fe_3_O_4_ nanocomposite was synthesized based on a chemical coprecipitation method [48] as follows: 0.5 g of GO powder was added to 100 mL of distilled water at 70°C and stirred using a magnetic magnet. Then, a mixture of 16.0 g of FeCl_3_.6H_2_O and 0.8 g of FeCl_2_.4H_2_O was added to the above mixture at 70°C. Then, the pH of the resulting suspension was adjusted to 12 by ammonia solution. The mixture was stirred for 1 hour at 70°C. Then, the resulting black precipitate, after cooling, was washed three times with distilled water and ethanol and dried for 6 hours at 60°C. In the second step, 2.0 g of GO-Fe_3_O_4_ nanocomposite was added to 400 mL of distilled water and stirred at 8°C for 30 min. Afterward, 40 mL of silicate solution (1.0 M) resulted from a 2-hour reflux of 4.22 g of SiO_2_, 2.0 g of NaOH, and40 mL of distilled water at 80°C was added into the initial solution. The pH of the resulting solution was adjusted to 6.0 using HCl and refluxed at 80°C for 6 hours. The resulting precipitate was isolated using magnets and washed with distilled water and dried at 60°C for 2 hours.

#### 3.2.2. Fabrication of MIP/GO-Fe_3_O_4_@SiO_2_ Nanocomposite

For synthesis of MIP/GO-Fe_3_O_4_@SiO_2_ nanocomposite, 0.6 g GO-Fe_3_O_4_@SiO_2_ nanocomposite was dispersed in 5 mL of acetonitrile for 10 min. Then, 0.012 mL of pyrrole, as a functional monomer, and 0.0006 g of MEL, as a template molecule, were added to the above suspension. Then, 0.3 g FeCl_3_, as the initiator, was added and was stirred in 300 r.p.m for 12 hours. Afterward, the black precipitate was collected using a magnet and was washed with deionized water. Then, the initiator removal test was performed by potassium thiocyanate. To remove MEL from the polymer structure, the black solid polymer produced by the 2 : 8 (V/V) acetic acid: methanol solvent was thoroughly washed until the absorbance of the extracted solution at 238 nm wavelength UV reached less than 0.005. After that, MIP/GO-Fe_3_O_4_@SiO_2_ nanocomposite was dried at 60°C for 2 hours. For nonimprinted polymer (NIP) preparation, polymerization was carried out in the absence of MEL.

#### 3.2.3. Fabrication of MIP/GO-Fe_3_O_4_@SiO_2_/Pt Electrode

To make the MIP/GO-Fe_3_O_4_@SiO_2_/Pt electrode, 0.07 g of nanocomposite was dispersed in 1.0 mL ethanol and was contacted with the surface of the Pt electrode. The electrode was dried at room temperature. Then, the resulting electrode was used as the working electrode in the electrochemical measurements.

### 3.3. Electroanalytical Measurements

The voltammetric measurements were done in a three-electrode system in the 0.3 M solution of [Fe (CN)_6_]^4−^ and [Fe (CN)_6_]^3−^. CV cycles were recorded from −0.5 V to +0.5 V at the scan rate of 8.0 mV/s, applying a step potential of 0.00405 V and modulation amplitude of 0.4995 V, at room temperature.

### 3.4. Sample Preparation

To evaluate the accuracy of the proposed method, 1.0 g food sample (milk, yoghurt, cheese, and dough) was spiked with a MEL standard solution to give a working concentration of MEL (0.0, 2.0, and 4.0 *μ*M). This sample was placed into a 5 Eppendorf Safe-Lock microcentrifuge tubes, including 4 mL of acetonitrile, then vortexed for 10 s, and finally was sonicated for 30 minutes. After that, the mixture was centrifuged at 2500 r.p.m. for 5 minutes to eliminate serum protein. The clear solution was transferred into a volumetric flask and fixed to 10 mL using deionized water [49].

## 4. Conclusion

In this study, an electrochemical sensor was developed for the determination of MEL. Chemical polymerization of pyrrole was carried out in the presence of MEL, and then, by removing the MEL from the MIP holes, it deposited on the bare Pt electrode to construct the electrochemical sensor as a selective and sensitive microsolid-phase preconcentration sensor for the determination of MEL. Screening of effective factors and their optimization was performed with multivariate optimization methods. The sensor was used for analysis of milk, yoghurt, cheese, and dough samples. It is noteworthy that the quick and easy-to-make renewal of the electrode, short incubation time, the characteristics of low detection limit (0.028 *µ*M), wide range (5.0 × 10^−7^ to 1.0 × 10^−5^ M), good repeatability (RSD 2.15%) and reproducibility (RSD 6.43%), simple fabrication, and low cost are the predominant advantages of the proposed sensor over the other existing methods of MEL analysis. The comparison between the analytical characteristics of the present sensor and some pervious reported technique for the determination of MEL is listed in Table 6 [7, 50, 51].

## Figures and Tables

**Table 1:** Results of PB experimental design matrix.

Run order	A	B	C	D	E	F	G	H	J	∆*I* (*μ*A)
1	0.6	15	Acetonitrile	0.3	150	0.005	24	500	10	21.71
2	0.6	25	Methanol	0.8	150	0.005	12	500	10	19.53
3	0.2	25	Acetonitrile	0.3	450	0.005	12	200	10	19.70
4	0.6	15	Acetonitrile	0.8	150	0.05	12	200	5	18.80
5	0.6	25	Methanol	0.8	450	0.005	24	200	5	19.64
6	0.6	25	Acetonitrile	0.3	450	0.05	12	500	5	19.79
7	0.2	25	Acetonitrile	0.8	150	0.05	24	200	10	18.67
8	0.2	15	Acetonitrile	0.8	450	0.005	24	500	5	21.41
9	0.2	15	Methanol	0.8	450	0.05	12	500	10	19.81
10	0.6	15	Methanol	0.3	450	0.05	24	200	10	21.37
11	0.2	25	Methanol	0.3	150	0.05	24	500	5	18.63
12	0.2	15	Methanol	0.3	450	0.005	12	200	5	18.76

**Table 2:** The CCD matrix and the experimental results.

Run order	B	E	G	∆*I* (*μ*A)
1	10	300	0.03	20.43
2	20	300	0.03	23.38
3	10	600	0.03	20.32
4	20	600	0.03	26.60
5	10	300	0.07	29.22
6	20	300	0.07	22.17
7	10	600	0.07	26.01
8	20	600	0.07	21.87
9	15	450	0.05	22.78
10	15	450	0.05	22.84
11	15	450	0.05	25.46
12	15	450	0.05	26.11
13	10	450	0.05	25.01
14	20	450	0.05	27.77
15	15	300	0.05	25.73
16	15	600	0.05	25.63
17	15	450	0.03	25.32
18	15	450	0.07	25.38
19	15	450	0.05	25.73
20	15	450	0.05	25.87

**Table 3:** The ANOVA results for evaluation of mathematical models obtained by response surface design.

Source	DF^a^	Adj. SS^b^	Adj. MS^c^	*F* value	*P* value
Linear	3	13.136	4.3788	59.49	0.001
Square	3	27.170	9.1495	124.30	0.001
Interaction	3	62.467	20.8224	282.89	0.001
Lack-of-fit	5	0.524	0.1049	2.48	0.171
Pure error	5	0.212	0.0424		
Total	19	103.788			

^a^Degrees of freedom; ^b^adjusted sum of squares; ^c^adjusted mean squares.

**Table 4:** Selectivity of sensor MEL (2.0 × 10^−6^ M) in presence of interfering molecules.

Interfering molecule	MEL : interfering molecule	Change in current response for detection of 2.0 × 10^−6^ M MEL	Recovery (%)
Arginine	1 : 1	−0.421	88.16
	1 : 2	−0.133	96.27
	1 : 4	−0.224	93.72
Galactose	1 : 1	+0.039	101.10
	1 : 2	−0.309	91.33
	1 : 4	−0.338	90.50
Glucose	1 : 1	−0.100	97.19
	1 : 2	−0.234	93.43
	1 : 4	+0.098	102.77
Maltose	1 : 1	−0.462	87.02
	1 : 2	−0.322	90.96
	1 : 4	−0.257	92.79
Rabeprazole	1 : 1	−0.230	93.54
	1 : 2	−0.328	90.80
	1 : 4	−0.421	88.16
Fluvoxamine	1 : 1	−0.261	92.65
	1 : 2	−0.002	99.41
	1 : 4	+0.260	107.31

**Table 5:** The results of MEL determination in food sample analysis.

	MEL added (*µ*M)	Average of MEL found (*µ*M)	Recovery (%)
Milk	0	Not detected	−
	2.0	2.09	104.5
Yoghurt	0	Not detected	−
	2.0	2.03	101.5
Cheese	0	Not detected	−
	2.0	1.85	92.5
Dough	0	Not detected	−
	2.0	1.95	95.0

**Table 6:** Comparison of the results of different techniques on determination of MEL.

Technique	Detection method	Linear range (M)	LOD (*µ*M)	Reference
HPLC	UV	7.9 × 10^−6^ − 6.3 × 10^−4^	0.79	[7]
Ion-selective electrode	Potentiometry	5.0 × 10^−6^ − 1.0 × 10^−2^	1.6	[50]
Gas chromatography	Mass spectrometry	4.0 × 10^−7^ − 1.58 × 10^−5^	0.08	[51]
MIP	CV	5.0 × 10^−7^ − 1.0 × 10^−5^	0.0028	This work

## Data Availability

No data were used to support this study.
